# Walnut Supplementation Restores the SIRT1-FoxO3a-MnSOD/Catalase Axis in the Heart, Promotes an Anti-Inflammatory Fatty Acid Profile in Plasma, and Lowers Blood Pressure on Fructose-Rich Diet

**DOI:** 10.1155/2021/5543025

**Published:** 2021-04-21

**Authors:** Maja Bošković, Maja Živković, Goran Korićanac, Jelena Stanišić, Manja Zec, Irena Krga, Aleksandra Stanković

**Affiliations:** ^1^VINČA Institute of Nuclear Sciences, National Institute of the Republic of Serbia, Laboratory for Radiobiology and Molecular Genetics, University of Belgrade, Belgrade, Serbia; ^2^VINČA Institute of Nuclear Sciences, National Institute of the Republic of Serbia, Laboratory for Molecular Biology and Endocrinology, University of Belgrade, Belgrade, Serbia; ^3^Center of Research Excellence in Nutrition and Metabolism, Institute for Medical Research, National Institute of the Republic of Serbia, University of Belgrade, Belgrade 11000, Serbia

## Abstract

The benefits of walnut (*Juglans regia*) consumption for metabolic health are known, but the molecular background underlying their putative antioxidant and anti-inflammatory/immunomodulatory effects is underexplored. We assessed that walnut supplementation (6 weeks) reverted unfavorable changes of the SIRT1/FoxO3a/MnSOD/catalase axis in the heart induced by fructose-rich diet (FRD). Intriguingly, Nox4 was increased by both FRD and walnut supplementation. FRD increased the cytosolic fraction and decreased the nuclear fraction of the uniquely elucidated ChREBP in the heart. The ChREBP nuclear fraction was decreased in control rats subjected to walnuts. In addition, walnut consumption was associated with a reduction in systolic BP in FRD and a decrease in fatty acid AA/EPA and AA/DHA ratios in plasma. In summary, the protective effect of walnut supplementation was detected in male rats following the fructose-induced decrease in antioxidative/anti-inflammatory capacity of cardiac tissue and increase in plasma predictors of low-grade inflammation. The current results provide a novel insight into the relationship between nutrients, cellular energy homeostasis, and the modulators of inflammatory/immune response in metabolic syndrome, emphasizing the heart and highlighting a track for translation into nutrition and dietary therapeutic approaches against metabolic disease.

## 1. Introduction

An increase in total fructose consumption over the past several decades has been correlated with metabolic syndrome (MetS) [1] and chronic inflammation [2]. The state of chronic inflammation in MetS can be described as a tissue-level stress response named “parainflammation” characterized by low-grade chronic activation of the immune system [3]. Maladaptive immune and inflammatory responses lead to systemic and cardiovascular insulin resistance [4], which are linked with MetS, cardiovascular disease (CVD) [5], and elevated oxidative stress [6, 7].

The role of nuts in the improvement of metabolic health, weight management, and glucose/insulin homeostasis markers and prevention of insulin resistance, hyperinsulinemia, dyslipidemia, and hypertension, the main characteristics of MetS, was suggested but studies are quite rare [8, 9]. In the protection of CVD, walnuts have the leading role [10], with the highest amount of a polyunsaturated omega-3 fatty acids (n-3 PUFAs), which have antioxidant and anti-inflammatory/immunomodulatory effects on the heart [11, 12]. They can be easily used in nutrition and could be essential for use in low-marine food intake countries. It has been shown that several different n-3 PUFA derivatives, docosahexaenoic acid (DHA), and alpha-linolenic acid (ALA) decrease ROS and NO production in LPS-stimulated macrophages [13, 14]. On the other hand, omega-6 fatty acids (arachidonic acid (AA)) are the precursor of inflammatory agents such as PGE2, cytokines, and interleukins and may have proinflammatory effects [15]. An unbalanced omega-6/omega-3 ratio in favor of omega-6 PUFAs contributes to MetS-related diseases [16] and have effects on immune cells [11]. An elevated AA/eicosapentaenoic acid (EPA) ratio represents an inflammatory biomarker in certain chronic diseases [17]; however, its capacity related to MetS-related diseases and diverse nutritional treatments is not fully understood.

Recent research suggests a strong link between the cellular energy homeostasis and immune cell activation and triggering of inflammation in response to cell and tissue damage [18]. It is supported by recent findings of the AMP-activated protein kinase (AMPK) role in inflammation and immunity [19], through AMPK repression of aerobic glycolysis and activation of mitochondrial oxidative metabolism. This role of AMPK in the inhibition of oxidative stress and inflammation seems to be closely related to a group of fuel-sensing molecules, the sirtuins, both involved in metabolic syndrome-associated diseases [20]. AMPK and sirtuin 1 (SIRT1) activate each other, suggesting the existence of an AMPK-SIRT1 cycle that links the cell's energy and redox states [20]. SIRT1 deacetylates the transcriptional regulator, forkhead box O3 (FoxO3a), thereby promoting the initiation of FoxO3a-dependent gene transcription [21] and upregulation of the expression of genes involved in antioxidative defence, such as mitochondrial manganese superoxide dismutase (MnSOD) and catalase [22, 23]. Recent studies have established a central role of SIRT1 in the relationship of the immune response to metabolism that are closely dependent on each other. SIRT1-mediated regulation of cellular metabolism reprogramming [24] is closely linked to a SIRT1 role in the appropriate dendritic cell function and innate immunity. Namely, the absence of SIRT1 alters the mitochondrial function and metabolic phenotype, resulting in the induction of fatty acid synthesis and leading to dysregulation of innate and adaptive immunity [25]. Recent findings [26] support a role of SIRT1 in the switch from early to late inflammation. SIRT1 inactivates NADPH oxidase 4 (Nox4) in cardiomyocytes [27] and rat aorta [28], which is of utmost importance for cardiomyocytes, as Nox4 is the major source of mitochondrial oxidative stress [29]. SIRT1 could be repressed by direct binding of the carbohydrate response element-binding protein (ChREBP) to its promoter, in metabolic active tissues [30]. ChREBP regulates gene transcription in glycolysis/fructolysis and de novo lipogenesis [31], which suggests its important role in the pathogenesis of metabolic diseases. Recent data [32] establish a critical role for ChREBP in preventing macrophage inflammation and apoptosis in atherosclerosis and demonstrate the importance of immune metabolic flux in chronic inflammatory diseases.

Based on the pointed relationship between mitochondria metabolism and inflammation/immunity and our previous findings [33] regarding fructose effects on target molecules in the heart, we hypothesized that walnut-enriched diet should have the capacity to revert the fructose-rich diet-induced changes in (1) metabolic stress axis mitochondrial molecule SIRT1-FoxO3a-MnSOD/catalase in the rat heart and Nox4 and ChREBP, as its unfavorable regulators, and (2) harmful ratios of proinflammatory/anti-inflammatory plasma PUFAs (omega-6/omega-3 fatty acid ratio), to achieve cardioprotective effects. This work will give a new insight into the relationship between nutrients, cellular energy homeostasis, and the modulators of the inflammatory/immune response in MetS, emphasizing that the heart as the molecular background of walnut supplementation in metabolic disorders has not been fully addressed, yet.

## 2. Materials and Methods

### 2.1. Walnut Characterization

Walnuts (*Juglans regia*) used in this study were purchased from a local market. A recent study described the complete fatty acid, macronutrient, and mineral composition of the walnuts [34]. In terms of fatty acid content, the most abundant n-3 fatty acid in the walnuts was ALA (C18:3 *n*-3, 11.2%), while linoleic acid (C18:2 *n*-6, 63.2%) was the most abundant n-6 fatty acid identified by gas chromatography, similar to the data reported in the European Food Information Resource [35].

### 2.2. Animal Model and Treatment

Twenty-one-day-old male Wistar rats were randomly divided into two groups according to diet regime—control group (C) (*n* = 18), with free access to tap water and standard commercial rat chow, and the fructose-fed group (F) (*n* = 18), with free access to the same food and 10% (*w*/*v*) fructose solution instead of tap water. Rats were housed in individual cages (3 rats per cage) and maintained under standard temperature (22°C) and 12 h light/dark cycles. This diet regime lasted for nine weeks. After that period, half of the control (CW) and fructose-fed rats received 2.4 g of dietary walnuts daily (FW), an amount which corresponds to half of the kernel. To ensure the original PUFA content in the walnuts, they were given to animals as whole kernels ensuring that they were eaten by each animal. After six weeks of this diet regime, all rats were sacrificed by decapitation and their hearts were removed, washed in saline, and stored at −70°C until analysis, while blood samples were collected in EDTA-containing tubes for biochemical measurements. A total of 36 animals participated in the experiment (9 animals per group), corresponding to the standards of statistics and ethics. Experimental protocols were approved by the Ethical Committee of the “Vinča” Institute of Nuclear Sciences for the Use of Laboratory Animals and performed in accordance with the guidelines of Directive 2010/63/EU of the European Parliament.

### 2.3. Feeding Behavior, Metabolic Parameters, and Blood Pressure of Experimental Animals

Food intake and liquid intake were recorded daily, while body mass was recorded weekly during the study period. Energy intake from the standard food, fructose solution, and walnuts was calculated and expressed as daily intake in kJ per rat.

The heart mass was weighted after removing from the body (absolute) and expressed relative to total body mass. Systolic blood pressure (SBP) and diastolic blood pressure (DBP) as well as heart rate frequency (HRF) were measured after dietary intervention in conscious rats by a noninvasive, computerized tail-cuff method BP system (Rat Tail Cuff Method Blood Pressure Systems (MRBP-R), IITC Life Science Inc., USA) with external preheating [36]. Mean arterial pressure (MAP), the average pressure in arteries during one cardiac cycle, was calculated as DBP plus 1/3 (SBP minus DBP). Pulse pressure (PP) was calculated as the difference between SBP and DBP.

### 2.4. Fatty Acid Analysis in Total Plasma Lipids

Blood samples were collected in EDTA tubes following overnight fasting, and fatty acids were analyzed as described before [37]. Briefly, plasma was separated by centrifugation (1600 × *g*, 10 min), aliquoted, and stored at −20°C until analysis. Total plasma lipids were extracted by the method of Folch et al. [38] using a chloroform-methanol mixture (2 : 1) with 0.05% (*w*/*v*) butylated hydroxytoluene. Fatty acids were esterified to methyl esters which were reconstituted in hexane and separated by gas chromatography on the Shimadzu chromatograph (GC-2014, Kyoto, Japan) equipped with a flame ionization detector and an RTX 2330-fused silica gel capillary column (60 m × 0.25 mm id × 0.2 *μ*m film thickness) (Restek Co., Bellefonte, PA, USA). For the purposes of the current study, arachidonic acid (AA) (20 : 4 *n*-6), eicosapentaenoic acid (EPA) (20 : 5 *n*-3), and docosahexaenoic acid (DHA) (22 : 6 *n*-3) were identified by comparing the peak retention times with a PUFA-2 standard mixture and Supelco 37 Component FAME Mix (Supelco Inc., Bellefonte, PA, USA). The amounts of individual fatty acids in plasma were presented as a percentage of the total identified fatty acids in the total lipid pool.

### 2.5. Cardiac Lysate Preparation

Cardiac tissue from three animals of the same group was pooled to prepare lysate, cytosolic, and nuclear fractions. Frozen pooled hearts were thawed and homogenized on ice with an Ultra-Turrax Homogenizer in modified RIPA buffer (pH 7.4) containing 50 mM Tris–HCl, pH 7.4, 150 mM NaCl, 1% Triton X-100, 0.2% Na-deoxycholate, 0.2% SDS, 1 mM EDTA, pH 7.4, protease inhibitors (1 mM PMSF, 10 *μ*g/mL leupeptin, and 10 *μ*g/mL aprotinin), and phosphatase inhibitors (1 mM activated sodium orthovanadate and 10 mM sodium fluoride). The homogenates were centrifuged at 15000 × *g* for 30 min at 4°C. Supernatants were boiled in Laemmli sample buffer and used as a cardiac cell lysate for Western blot analysis.

### 2.6. Preparation of Cytosolic and Nuclear Fractions

The rest of the total pooled heart weight was homogenized on ice with an Ultra-Turrax Homogenizer in homogenization TEMG buffer (pH 7.5) containing 50 mM Tris–HCl, 1 mM EDTA, 12 mM monothioglycerol, 10% glycerol (*v*/*v*), protease, and phosphatase inhibitors. The cell lysate was filtered through gauze and centrifuged (1000 × *g*, 15 min). Cytosolic proteins were isolated from the original supernatant (Sn1), while nuclear proteins were isolated from the original pellet. The original supernatant (Sn1) was centrifuged at 12000 × *g* for 30 min. The upper phase containing cytosolic proteins was collected by centrifugation of the supernatant (Sn2) at 100000 × *g* for 1 h. The original pellet was washed twice in TMG + 0.2% Triton X-100 buffer and once in TMG buffer, pH 7.5 (20 mM Tris–HCl, 12 mM monothioglycerol, and 10% glycerol (*v*/*v*)), followed by centrifugation (1000 × *g*, 15 min) after each wash. The resulting pellet was resuspended in TEMG + 0.5 M KCL buffer and incubated on ice for 1 h with frequent vortexing. The supernatant containing nuclear proteins was collected by centrifugation of this resuspended pellet (34 200 rpm, 1 h). Samples were boiled in Laemmli sample buffer and used as a cardiac cytosolic and nuclear fraction for Western blot analysis.

### 2.7. Western Blot Analysis

Cardiac proteins (50 *μ*g/lane) were separated by electrophoresis on 10% SDS polyacrylamide gels and transferred to polyvinylidene fluoride (PVDF) membranes. The uniformity of protein loading in each lane was assessed by staining the membranes with Ponceau S (Sigma-Aldrich, P3504), and this total protein staining quantified by ImageJ software was used as a loading control [39]. After destaining, membranes were blocked with 5% (*w*/*v*) milk in TBST and incubated with primary antibody for AMPK (sc-25792), SIRT1 (sc-74465), FoxO3a (sc-11351), MnSOD (sc-30080), catalase (ab16731), Nox4 (ab133303), or ChREBP (NB400-135) overnight. Unbound and nonspecifically bound antibodies were washed with TBST, and membranes were incubated with horseradish peroxidase- (HRP-) conjugated secondary anti-rabbit antibody (for AMPK, FoxO3a, MnSOD, catalase, and ChREBP antibody) or anti-mouse antibody (for SIRT1 antibody) (Santa Cruz Biotechnology) for 1.5 h at room temperature. After washing, the proteins were visualized by the enhanced chemiluminescence (ECL) method. The films were scanned, and quantitative analyses based on densitometry of protein bands on X-ray film were performed by ImageJ software (NIH, USA). As AMPK, FoxO3a, and ChREBP shuttle between the nucleus and the cytoplasm, protein levels of their total forms were determined in the cytosolic and nuclear fractions of cardiac tissue. The levels of SIRT1, MnSOD, catalase, and Nox4 were determined in the cardiac cell lysate. All Western blot experiment results were expressed as the protein/total protein staining ratio. The levels of proteins are presented as a fold of the appropriate control value.

### 2.8. Statistical Analysis

Statistical analysis was performed using Statistica software package. Data were expressed as mean ± standard deviation (SD) for 9 animals per experimental group (a total of 36 animals). The results were analyzed using two-way analysis of variance (ANOVA) (evaluating fructose and walnut factors, as well as mutual interactions), followed by the Tukey's post hoc test to evaluate differences between groups. A value of *p* < 0.05 was considered as statistically significant.

## 3. Results

### 3.1. Relative Heart Mass, Blood Pressure, and Heart Rate Frequency in Experimental Treatments

We calculated the energy intake in kJ/day/rat and expressed intake from each source (chow food, fructose solution, and walnut) as a percentage of the total. Control animals had 100% chow energy intake. The total energy intake of CW rats was the sum of chow energy intake (90.66%) and walnut energy intake (9.34%); while in the F group, it was the sum of the chow energy intake (63.27%) and liquid energy intake (36.73%). The FW group had a total energy intake from all three sources: chow energy intake (59.13%), liquid energy intake (33.43%), and walnut energy intake (7.44%). There was no significant difference in liquid energy intake in the F and FW groups yet; as previously published in the current animal model, the significant main effect of fructose (*p* < 0.001) and walnut supplementation (*p* < 0.01) on total energy intake was detected [40].

We detected significant main effects of walnut supplementation on relative heart mass (two-way ANOVA, *p* < 0.01), but post hoc comparisons showed a significant difference only between control animals and combined fructose and walnut treatment (*p* = 0.04) (Table 1). Total body mass at the end of the study was affected by both factors, FRD (*p* < 0.01) and walnut supplementation (*p* < 0.001).

We also demonstrated the significant main effects of walnut supplementation and fructose × walnut interaction (*p* < 0.01, partial eta-squared 0.45, observed power 0.93 for alpha = 0.05) on systolic blood pressure, while these factors did not show any significant effect on diastolic blood pressure (Table 1). Walnut supplementation gained the significant main effect on the heart rate frequency (*p* < 0.01) (Table 1). Post hoc test revealed no effect of walnut-enriched diet on blood pressure and heart rate frequency of control rats; but in fructose-fed rats, walnut supplementation was associated with a significant reduction in systolic blood pressure (F vs FW, *p* < 0.01, and C vs FW, *p* < 0.05) and showed trend toward a decrease in heart rate frequency (F vs FW, *p* = 0.08). However, fructose and walnut consumption and their interaction did not have a significant effect on either MAP or PP.

### 3.2. Effects of Fructose-Rich Diet and Walnut Supplementation on AA/EPA and AA/DHA in Total Plasma Lipids

Our results showed that the intake of walnuts reversed the fructose-induced increase in AA/EPA (*p* for interaction < 0.001, partial eta-squared 0.39, and observed power 0.99 for alpha = 0.05). On the other hand, regardless of the metabolic burden associated with fructose intake, walnut supplementation decreased AA/DHA levels (*p* = 0.013, Table 2).

### 3.3. Effects of Fructose-Rich Diet and Walnut Supplementation on the Protein Content of AMPK-SIRT1-FoxO3a-MnSOD/Catalase, ChREBP, and Nox4 in the Heart of Male Rats

**Figure 1:**
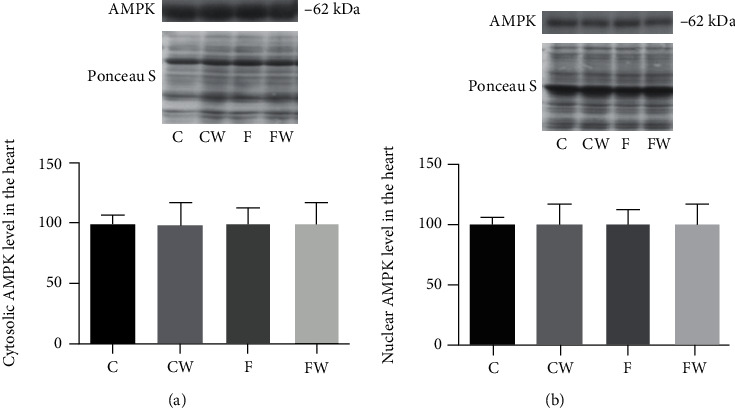
Effects of fructose-rich diet and supplementation with walnuts on the (a) cytosolic and (b) nuclear AMPK protein levels in the heart of experimental rats. Values are means with standard deviations represented by vertical bars for 9 animals per group. C: animals on standard laboratory chow; F: animals fed a fructose-rich diet; CW: animals on standard laboratory chow and walnut supplementation; FW: fructose-fed animals on walnut supplementation.

Our results did not show any significant effect of fructose/walnut supplementation or fructose × walnut interaction on the cytosolic or nuclear AMPK protein level in the rat heart (Figures 1(a) and 1(b)).

**Figure 2:**
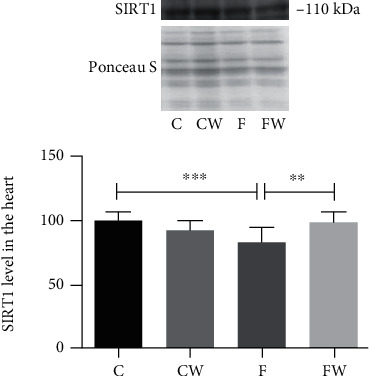
Effects of fructose-rich diet and supplementation with walnuts on the SIRT1 protein level in the heart of experimental rats. Values are means with standard deviations represented by vertical bars for 9 animals per group. C: animals on standard laboratory chow; F: animals fed a fructose-rich diet; W: animals fed a dietary walnut; CW: animals on standard laboratory chow and walnut supplementation; FW: fructose-fed animals on walnut supplementation; ^∗∗^*p* < 0.01; ^∗∗∗^*p* < 0.001.

The results showed the significant main effects of fructose (*p* < 0.05) and fructose × walnut interaction (*p* < 0.001, partial eta-squared 0.30, and observed power 0.98 for alpha = 0.05) on the SIRT1 protein level (Figure 2). FRD significantly decreased the SIRT1 protein level in rat hearts compared to control animals (C vs F, *p* < 0.001), while the walnut supplementation of fructose-fed animals elevated its level compared to rats fed only fructose (F vs FW, *p* < 0.01).

**Figure 3:**
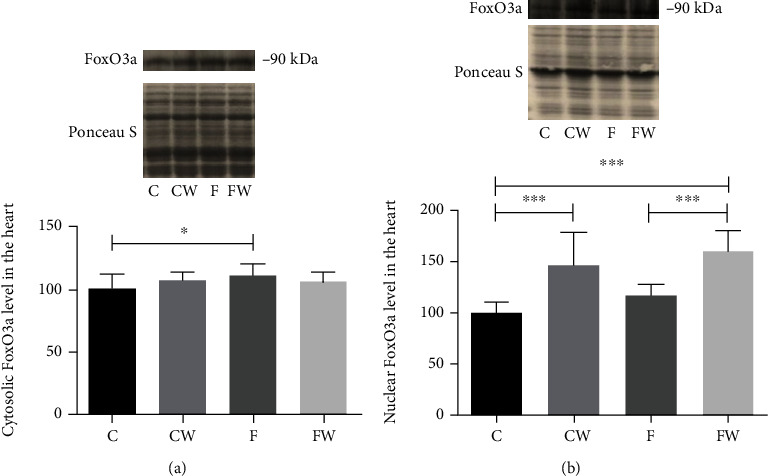
Effects of fructose-rich diet and walnuts on the (a) cytosolic and (b) nuclear FoxO3a protein levels in the heart of experimental rats. Values are means with standard deviations represented by vertical bars for 9 animals per group. C: animals on standard laboratory chow; F: animals fed a fructose-rich diet; CW: animals on standard laboratory chow and walnut supplementation; FW: fructose-fed animals on walnut supplementation; ^∗^*p* < 0.05; ^∗∗∗^*p* < 0.001.

The results demonstrated a significant effect of fructose × walnut interaction on the cytosolic FoxO3a protein level (*p* < 0.05, partial eta-squared 0.10, and observed power 0.54 for alpha = 0.05) and significant main effects of fructose (*p* < 0.05) and walnut supplementation (*p* < 0.001) on the nuclear FoxO3a protein level (Figures 3(a) and 3(b)). FRD significantly increased the cytosolic FoxO3a protein level (C vs F, *p* < 0.05), while walnut supplementation elevated the nuclear FoxO3a protein level in the hearts of both, control (C vs CW, *p* < 0.001) and fructose-fed rats (F vs FW, *p* < 0.001, and C vs FW, *p* < 0.001, respectively).

**Figure 4:**
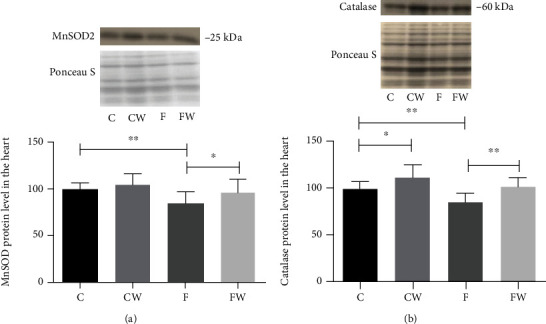
Effects of fructose-rich diet and walnuts on the (a) MnSOD and (b) catalase protein levels in the heart of experimental rats. Values are means with standard deviations represented by vertical bars for 9 animals per group. C: animals on standard laboratory chow; F: animals fed a fructose-rich diet; CW: animals on standard laboratory chow and walnut supplementation; FW: fructose-fed animals on walnut supplementation; ^∗^*p* < 0.05; ^∗∗^*p* < 0.01.

Significant main effects of fructose and walnut supplementation on the MnSOD (*p* < 0.001 and *p* < 0.01, respectively) and catalase (*p* < 0.001 and *p* < 0.001, respectively) protein levels were detected (Figures 4(a) and 4(b)). FRD decreased the MnSOD and catalase protein levels (C vs F, *p* < 0.01, for both), while walnut supplementation reverted these changes (F vs FW, *p* < 0.05 and *p* < 0.01, respectively). Walnut-enriched diet significantly increased the catalase level even in control animals (C vs CW, *p* < 0.05).

**Figure 5:**
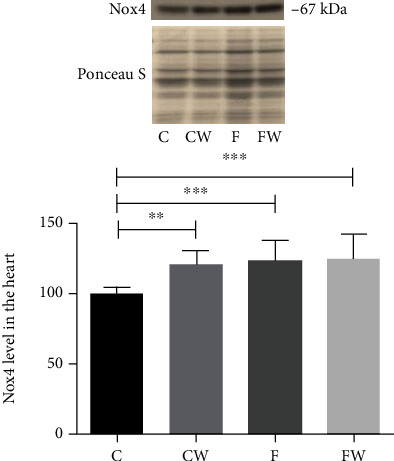
Effects of fructose-rich diet and walnut supplementation on the Nox4 protein level in the heart of experimental rats. Values are means with standard deviations represented by vertical bars for 9 animals per group. C: animals on standard laboratory chow; F: animals fed a fructose-rich diet; CW: animals on standard laboratory chow and walnut supplementation; FW: fructose-fed animals on walnut supplementation; ^∗∗^*p* < 0.01; ^∗∗∗^*p* < 0.001.

The significant main effects of fructose (*p* < 0.001) and walnut supplementation (*p* < 0.01) as well as a significant effect of fructose × walnut interaction (*p* < 0.05, partial eta-squared 0.15, and observed power 0.72 for alpha = 0.05) on the Nox4 protein level were detected (Figure 5). Post hoc analyses revealed a significant increase in Nox4 protein expression after both FRD (C vs F, *p* < 0.001) and walnut supplementation (C vs CW, *p* < 0.01, and C vs FW, *p* < 0.001) compared to the control group.

**Figure 6:**
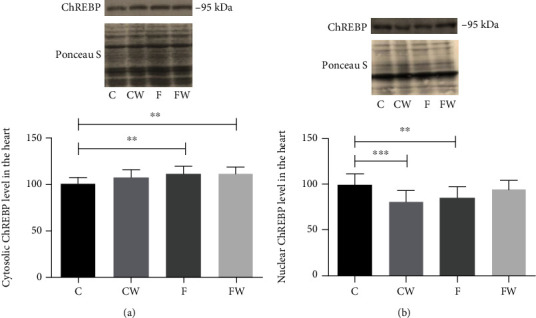
Effects of fructose-rich diet and walnut supplementation on the (a) cytosolic and (b) nuclear ChREBP protein levels in the heart of experimental rats. Values are means with standard deviations represented by vertical bars for 9 animals per each group. C: animals on standard laboratory chow; F: animals fed a fructose-rich diet; CW: animals on standard laboratory chow and walnut supplementation; FW: fructose-fed animals on walnut supplementation; ^∗∗^*p* < 0.01; ^∗∗∗^*p* < 0.001.

A significant main effect of fructose on the cytosolic ChREBP protein level (*p* < 0.01) as well as a significant effect of fructose × walnut interaction on the nuclear ChREBP protein level (*p* < 0.001, partial eta-squared 0.25, and observed power 0.99 for alpha = 0.05) was demonstrated (Figures 6(a) and 6(b)). Post hoc analyses revealed that FRD increased the ChREBP protein level in the cytosolic fraction (C vs F, *p* < 0.01) and decreased its level in the nuclear fraction compared to the control group (C vs F, *p* < 0.01). Walnut consumption significantly decreased the nuclear ChREBP protein level in control rats (C vs CW, *p* < 0.001) and showed a trend toward an increase in the cytosolic ChREBP protein level of control rats (C vs CW, *p* = 0.07). In the cytosolic fraction, walnut consumption significantly increased the ChREBP protein level in fructose-fed rats compared to the control group (C vs FW, *p* < 0.01).

## 4. Discussion

Among other nuts, walnuts could be promising for use in dietary supplementation as they contain high levels of n-3 PUFAs, dietary fiber, antioxidants, and phytosterols [10, 41]. Our study suggests a beneficial role of walnuts in the improvement of the metabolic status that is mainly impaired by high fructose intake, a common component of the modern lifestyle. In addition, we demonstrated an increased omega-6/omega-3 ratio in FRD rats, which has been suggested to be highly prothrombotic and proinflammatory and to contribute to the prevalence of atherosclerosis and MetS-related diseases [16, 42]. The tissue-specific omega-6/omega-3 decrease after walnut consumption in high-fructose-fed Wistar rats was recently demonstrated [37]. An increase in the AA/EPA ratio as a result of fructose feeding in our study could be deleterious for the heart; previously, it has been suggested that higher levels of the AA/EPA ratio are associated with a greater risk for CVD and has been proposed as a biochemical marker of CVD events [16, 42]. We demonstrated a decrease in the AA/EPA and AA/DHA ratio in plasma as a result of walnut consumption and walnut × fructose interaction, which confirms that walnut consumption could be beneficial in dietary regime especially in MetS and in the prevention of CVD associated with MetS. The additional beneficial effect of walnut consumption in our study was the reduction of systolic BP in FRD rats, another risk factor for CVD. In the literature, the effect of walnuts or other nuts on BP was inconsistent yet but our result is in line with studies that have found a reduction in BP [9, 43]. Effects of walnut consumption on BP lowering in FRD rats may be related to the rich cation content, such as magnesium and potassium [44] and high content of ALA [10], which induces coronary VSMC relaxation [45]. The observed effect of walnut consumption on BP could be in association with its beneficial effect on the vascular tone, through the ATP-sensitive potassium channel, recently presented in the same model of FRD rats [40].

The main results of our study about the beneficial effects of walnuts on the antioxidant AMPK-SIRT1-FoxO3a-MnSOD/catalase axis in the heart of fructose-fed male rats are in line with previous evidence that n-3 PUFAs induce significant cardiovascular benefits, which appear to be achieved via its antioxidant and anti-inflammatory/immunomodulatory effects on the heart [11, 12]. The decrease of the omega-6/omega-3 fatty acid ratio, which is in our study related to walnut consumption, has been previously associated with a decrease of circulating inflammatory markers [46, 47]. Several recent studies have connected inflammation with fatty acids as important providers of nutritional needs of immune cells after sensing metabolic stress [48]. Two nutrient sensors, AMPK and SIRT1, interact to inhibit oxidative stress and macrophage inflammation, which appear to be involved in the pathogenesis of MetS [20]. Decreased nuclear SIRT1 levels/activity increases NF-*κ*B activity and amplifies proinflammatory gene expression during chronic inflammation [49]. On the other hand, activation of SIRT1 by polyphenol fisetin [50] or by n-3 PUFA [51] was observed to be anti-inflammatory through inhibition of downstream expression of cytokines, chemokines, prostaglandins, and MMP-s. This guided us to investigate SIRT1 and AMPK in the heart of FRD rats, as well as the potential of walnuts to rebalance sirtuins as molecules that integrate metabolism, bioenergetics, and immunity during inflammation in MetS. Previously, we have detected that fructose could contribute to inflammation through the changes of MMP-9 expression that could be mediated via the NF-*κ*B pathway in the heart [2]. In addition, we showed that estrogen replacement exerted beneficial effects on the AMPK-SIRT1-FoxO3a-MnSOD/catalase axis in the heart of FRD rats, which has been compromised by fructose overload [33]. The effects of walnut supplementation on this axis and related molecules, Nox4 and ChREBP, in the heart of FRD rats have not been previously determined. We hypothesized that reduced oxidative stress in the heart, as a result of walnut supplementation, could be also mediated by suppression of ROS production by Nox4. It has been demonstrated that n-3 PUFA could disrupt Toll-like receptor 4- (TLR4-) induced activation of Nox [52] and inhibit ROS-dependent activation of the proinflammatory transcription factor NF-*κ*B in endothelial cells [53]. It was documented that a diet enriched with 60% fructose increased the levels of Nox4 mRNA in the rat heart and aorta [6]. In the current study, a diet enriched with 10% fructose increased the protein level of Nox4 in the rat heart. As previously denoted, Nox-mediated ROS production has a central role in vascular inflammatory responses during nutritional excess [54]. Vascular Nox production could be additionally enhanced by Ang II [55], which is the main effector molecule of the activated unfavorable arm of the RAS in the heart/aorta of a fructose-fed rat model [56]. Activated NADPH oxidase leads to the generation of ROS, which activate NF-*κ*B to increase the transcription of cytokines (TNF and IL-6). The binding of these cytokines to their sarcolemmal receptors induce serine kinases that phosphorylate IRS-1 and inhibit cardiac insulin signaling, providing a novel interaction path between Ang II and insulin signaling [57]. In the present study, elevated Nox4 protein levels induced by FRD contribute to inflammation in the heart, possibly through Ang II- and ROS-dependent mechanisms. However, recent data also suggested a possible atheroprotective role of Nox4 [58, 59], as well as its role in maintaining the cardiac energetic status [60]. In line with this, we could suggest that increased Nox4 protein levels in control animals after walnut supplementation, detected in our study, indicate its possible role in cardiovascular homeostasis and adaptation to chronic inflammatory stress. Even so, this should be further investigated.

While both FRD and walnut supplementation did not affect AMPK, they did significantly alter the SIRT1 protein level, indicating the possibility that SIRT1 expression in the heart is not regulated via AMPK, at least in current experimental conditions, which is in agreement with our previous study [33] and recent results in the aorta in the same FRD model [40]. Herein, FRD decreased the SIRT1 protein level in the rat heart, in line with the findings that MetS, obesity, and chronic inflammation have been associated with reduced levels of SIRT1 [61]. SIRT1-deficient *ob/ob* mice show exaggerated microvascular inflammation in comparison with lean mice [62]. In addition, SIRT1 deletion in myeloid cells increased the infiltration of M1 macrophages and decreased M2 macrophages in adipose tissue in mice on high-fat diets, resulting in insulin resistance [63] that is present in our FRD model. The studies of Yoshizaki et al. directly linked the anti-inflammatory effects of SIRT1 in adipocytes and macrophages with improved insulin sensitivity [64]. SIRT1 has been located in the nuclei of a significant fraction of cardiomyocytes [65] and could yield protection of cardiomyocytes. In alignment with our hypothesis, walnut supplementation restored the SIRT1 protein level in the FRD rat heart, indicating protection of the heart and a probable decrease of inflammation. The pluripotent regulatory role of sirtuins, which we previously discussed, leads to their recognition as new therapeutic targets. Thus, the determined effect of walnuts on SIRT1 represents the basis for further investigation of nutritional treatments regarding the metabolic state and inflammatory/immunomodulatory processes in the heart.

The downstream effects of SIRT1 include promotion of FoxO3a dephosphorylation and consequent initiation of FoxO3a-dependent gene transcription [28]. Our results suggest that a decrease in SIRT1 by FRD might underline the increased FoxO3a phosphorylation and downregulation of dependent genes. As FoxO3a regulates gene expression of MnSOD [22] and catalase [23], their decreased protein content in fructose-fed rats in the current study might be related to increased cytosolic FoxO3a in FRD. Importantly, our study revealed a significant increase in the nuclear protein level of FoxO3a following walnut supplementation, and consequently, we detected an increase in the MnSOD and catalase protein content in walnut-enriched diet groups.

Fructose activates ChREBP, which could repress SIRT1 expression in metabolic-active tissues [30], but data about the heart are missing. Moreover, in the rat liver, ChREBP activity was markedly higher in rats fed with high fructose compared with isocaloric high-glucose diets [66]. During fasting, the nuclear import and transactivity of ChREBP were inactivated [67] while these processes are activated by the products of carbohydrate metabolism [68]. In our study, however, FRD significantly increased the cytosolic and decreased nuclear ChREBP protein levels in the heart. The lack of studies dealing with ChREBP in the heart and the complexity of tissue-specific expression suggest probable differential expression of ChREBP in the heart compared to metabolic active tissues [31, 69]. It is feasible that SIRT1 expression in the heart is not regulated via ChREBP and that ChREBP is important to regulate gene transcription in glycolysis/fructolysis in tissues that preferentially oxidize carbohydrates, to provide ATP for energy-expensive metabolic processes [70]. Fatty acid oxidation provides 60–70% of the energy requirements in the heart [71], but fructose can also be used as an energy substrate in the heart, considering that cardiomyocytes express the fructose-specific GLUT5 transporter [72]. Our results suggest that ChREBP is also more prevalent in the cytosol after walnut supplementation of fructose-fed animals, indicating the suppression of ChREBP activity in the heart of walnut-fed rats. A decreased level of nuclear localization of ChREBP in the heart after walnut supplementation, similar to the previously shown in the liver on high-fat diet [67], could be due to elevated amounts of fatty acids in the heart. Fatty acid-rich diets have been shown to suppress ChREBP expression by accelerating ChREBP mRNA decay [67, 73] and inhibit the nuclear localization of ChREBP [73]. Decreased DNA-binding activity of ChREBP has been observed in rats fed a high-fat diet, compared to a high-carbohydrate diet, which also implicates ChREBP in the mechanism of fatty acid inhibition of glycolysis and lipogenesis [67, 69]. However, further results on the ChREBP in the heart should be provided to bring us closer to understand its role upon nutritional treatment.

## 5. Conclusions

The current study has confirmed the benefits of walnut consumption and suggested the mechanisms underlying their cardioprotective effects, emphasizing the role of SIRT-1 as the nutritional sensor that transmits molecular signals responsible for the metabolic state and inflammatory/immunomodulatory processes in the heart. The SIRT1-FoxO3a-MnSOD/catalase axis, compromised by fructose overload, was balanced upon walnut treatment. The anti-inflammatory effect of walnut fatty acids, expressed in the form of a decreased AA/EPA and AA/DHA ratio, is likely mediated through negative regulation of NF-*κ*B signaling and downstream cytokine expression, which should be investigated in the future study. Intriguing results of a decrease in nuclear ChREBP in the heart upon walnut supplementation, resembling changes in the metabolic tissue on high-fat diet, should be further deepened as the available data about this molecule in the heart are scarce. Nevertheless, this study provides novel data regarding the molecular background of anti-inflammatory and antioxidative effects of walnuts in the heart, which may be translated into modulation of nutrition and dietary therapeutic approaches against metabolic disease.

## Figures and Tables

**Table 1:** Effects of fructose-rich diet and walnut supplementation on total body mass, relative heart mass, and blood pressure in experimental rats.

	C	CW	F	FW	Two-way ANOVA		
					W	F	F × W
Total body mass (g)	463.22 ± 34.3	513.33 ± 36.54^∗^	485.44 ± 30.78	554.44 ± 33.22^∗∗∗,$$$^	<0.001	<0.01	NS
Heart mass/total body mass (×100)	0.25 ± 0.02	0.23 ± 0.02	0.24 ± 0.01	0.22 ± 0.03^∗^	<0.01	NS	NS
SBP (mm·hg)	147.4 ± 3.36	149.8 ± 3.49	150.8 ± 5.54	138.8 ± 4.97^∗,$$,##^	<0.05	NS	<0.01
DBP (mm·hg)	84.6 ± 6.8	91 ± 9.49	88.8 ± 13.81	84.6 ± 5.03	NS	NS	NS
HRF (beats/min)	359.6 ± 29.31	323.2 ± 29.52	390.4 ± 42.17	336.6 ± 27.02	<0.01	NS	NS
MAP (mm·hg)	103.17 ± 6.3	107.06 ± 10.21	106.06 ± 12.39	107.17 ± 9.33	NS	NS	NS
PP (mm·hg)	62.5 ± 9.89	58.17 ± 10.15	62.67 ± 10.98	54.50 ± 15.67	NS	NS	NS

Data are presented as mean ± SD for 9 animals per group. A value of *p* < 0.05 was considered as statistically significant. C: animals on standard laboratory chow; CW: animals on standard laboratory chow and walnut supplementation; F: animals fed a fructose-rich diet; FW: fructose-fed animals on walnut supplementation; SBP: systolic blood pressure; DBP: diastolic blood pressure; HRF: heart rate frequency; MAP: mean arterial pressure; NS: not significant. Significantly different from ^∗^control, ^#^walnuts, and ^$^fructose. Significance is ^∗^*p* < 0.05, ^##$$^*p* < 0.01, and ^∗∗∗$$$^*p* < 0.001.

**Table 2:** Effects of fructose-rich diet and walnut supplementation on the total plasma AA/EPA and AA/DHA ratio in experimental rats.

	C	CW	F	FW	Two-way ANOVA		
					F	W	F × W
AA/EPA	116 ± 58	87.09 ± 20.31	204.50 ± 46.87	58.92 ± 17.09	0.032	0.000	0.000
AA/DHA	15.17 ± 7.35	8.28 ± 2.61	12.36 ± 3.80	11.17 ± 2.35	0.977	0.013	0.071

Data are presented as mean ± SD for 9 animals per group. A value of *p* < 0.05 was considered as statistically significant. C: animals on standard laboratory chow; F: animals fed a fructose-rich diet; CW: animals on standard laboratory chow and walnut supplementation; FW: fructose-fed animals on walnut supplementation; AA: arachidonic acid (20 : 4 *n*-6); EPA: eicosapentaenoic acid (20 : 5 *n*-3); DHA: docosahexaenoic acid (22 : 6 *n*-3).

## Data Availability

Data are available upon request (contact the corresponding author).

## Supplementary Materials

Supplementary 1 Graphical abstract.

Supplementary 2 ARRIVE checklist.
