# Supplementary material for: Walnut Supplementation Restores the SIRT1-FoxO3a-MnSOD/Catalase Axis in the Heart, Promotes an Anti-Inflammatory Fatty Acid Profile in Plasma, and Lowers Blood Pressure on Fructose-Rich Diet
Source: Oxid Med Cell Longev. 2021 Apr 21;2021:5543025. doi: 10.1155/2021/5543025 (PMC8086433; doi:10.1155/2021/5543025)
Supplement: Supplementary 1 — Graphical abstract. [file 5543025.f1.docx]

NOTE: Please save this file locally before filling in the table, DO NOT work on the file within your internet browser as changes will not be saved. Adobe Acrobat Reader (available free [here](https://acrobat.adobe.com/uk/en/acrobat/pdf-reader.html)) is recommended for completion.


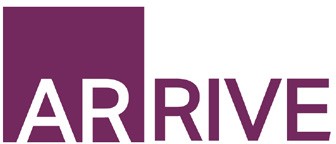
The ARRIVE guidelines 2.0: author checklist

| The ARRIVE Essential 10 | | | |
| --- | --- | --- | --- |
| These items are the basic minimum to include in a manuscript. Without this information, readers and reviewers cannot assess the reliability of the findings. | | | |
| **Item** |  | **Recommendation** | **Section/line number, or reason for not reporting** |
| **Study design** | 1 | For each experiment, provide brief details of study design including:   1. The groups being compared, including control groups. If no control group has been used, the rationale should be stated. 2. The experimental unit (e.g. a single animal, litter, or cage of animals). | Subsection Animal model and treatment |
| **Sample size** | 2 | 1. Specify the exact number of experimental units allocated to each group, and the total number in each experiment. Also indicate the total number of animals used. 2. Explain how the sample size was decided. Provide details of any *a priori* sample size calculation, if done. | a. 122  b.123 |
| **Inclusion and exclusion criteria** | 3 | 1. Describe any criteria used for including and excluding animals (or experimental units) during the experiment, and data points during the analysis. Specify if these criteria were established *a priori.* If no criteria were set, state this explicitly. 2. For each experimental group, report any animals, experimental units or data points not included in the analysis and explain why. If there were no exclusions, state so. 3. For each analysis, report the exact value of *n* in each experimental group. | a. All animals were healthy at entering the experiment and none were excluded from the experiment. No a priori exclusion criteria were established, based on our previous experience with use of the same fructose treatment.  b. There were no exclusion  c. For each analysis n=9 animals were included per group (total, 4 groups in the study) |
| **Randomisation** | 4 | 1. State whether randomisation was used to allocate experimental units to control and treatment groups. If done, provide the method used to generate the randomisation sequence. 2. Describe the strategy used to minimise potential confounders such as the order of treatments and measurements, or animal/cage location. If confounders were not controlled, state this explicitly. | a. Rats were allocated to the one of four experimental groups randomly. Allocation of more than one animal from the same litter has been avoided. Body weight, to ensure minimal differences among animals before entering the experiment, were used as additional criteria including the maximum match of date of birth, as well. All animals were confirmed to be healthy by veterinary, before entering the experiment. |
| **Blinding** | 5 | Describe who was aware of the group allocation at the different stages of the experiment (during the allocation, the conduct of the experiment, the outcome assessment, and the data analysis). | JS, GK-allocation, conduct of experiment (treatment), MB, GK, MZec-outcome assessment, MZ, AS-data analysis |
| **Outcome measures** | 6 | 1. Clearly define all outcome measures assessed (e.g. cell death, molecular markers,   or behavioural changes).   1. For hypothesis-testing studies, specify the primary outcome measure, i.e. the outcome measure that was used to determine the sample size. | a. Molecular markers  b. Plasma levels of arachidonic acid, - eicosapentaenoic acid docosahexaenoic acid to confirm that sample size is relevant for attaining the statistical significance of <0.05 with at least 80% of probability |
| **Statistical methods** | 7 | 1. Provide details of the statistical methods used for each analysis, including software used. 2. Describe any methods used to assess whether the data met the assumptions of the statistical approach, and what was done if the assumptions were not met. | Subsection Statistical analysis |
| **Experimental animals** | 8 | 1. Provide species-appropriate details of the animals used, including species, strain and substrain, sex, age or developmental stage, and, if relevant, weight. 2. Provide further relevant information on the provenance of animals, health/immune   status, genetic modification status, genotype, and any previous procedures. | 111 |
| **Experimental procedures** | 9 | For each experimental group, including controls, describe the procedures in enough detail to allow others to replicate them, including:   1. What was done, how it was done and what was used. 2. When and how often. 3. Where (including detail of any acclimatisation periods). 4. Why (provide rationale for procedures). | Section Materials and Methods  110-195 |
| **Results** | 10 | For each experiment conducted, including independent replications, report:   1. Summary/descriptive statistics for each experimental group, with a measure of variability where applicable (e.g. mean and SD, or median and range). 2. If applicable, the effect size with a confidence interval. | Section Results |
