# Supplementary figures and images for: Walnut Supplementation Restores the SIRT1-FoxO3a-MnSOD/Catalase Axis in the Heart, Promotes an Anti-Inflammatory Fatty Acid Profile in Plasma, and Lowers Blood Pressure on Fructose-Rich Diet
Source: Oxid Med Cell Longev. 2021 Apr 21;2021:5543025. doi: 10.1155/2021/5543025 (PMC8086433; doi:10.1155/2021/5543025)

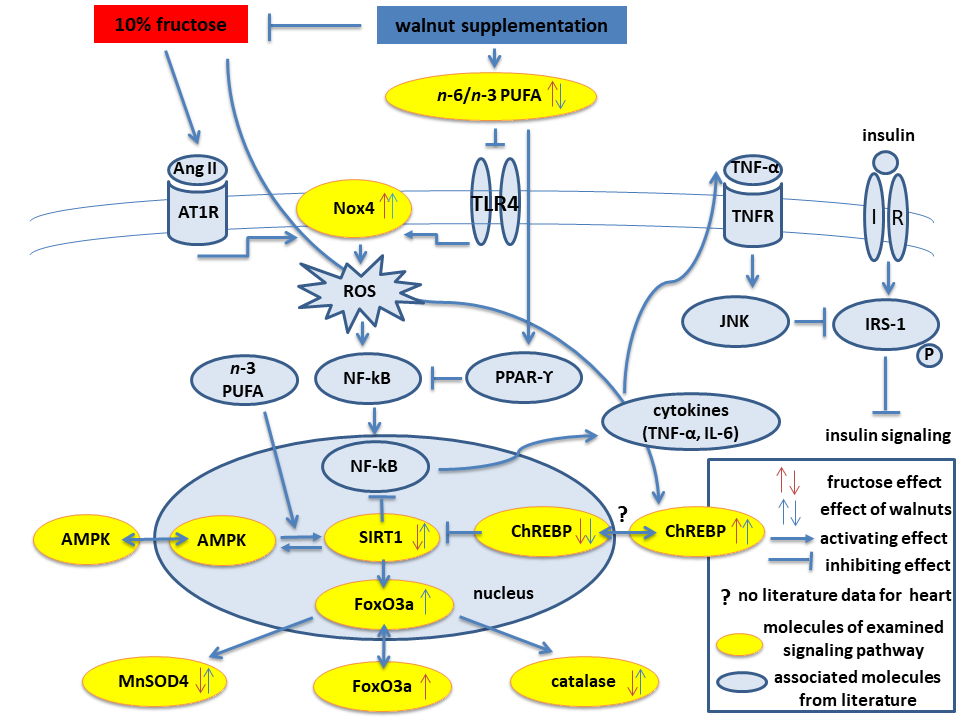

Supplement: Supplementary 2 — ARRIVE checklist. [file 5543025.f2.docx]
